# Healthcare accessibility since and during Covid-19 pandemic among older individuals in Europe: Madara Mikelsone

**DOI:** 10.1093/eurpub/ckac130.108

**Published:** 2022-10-25

**Authors:** M Mikelsone, I Reine

**Affiliations:** Statistics Unit, Riga Stradiņš University, Rīga, Latvia

## Abstract

Ensuring access to healthcare is critical to prevent illnesses and maintain health and functional abilities for older individuals. The Covid-19 pandemic is a major global public health threat that challenges healthcare availability and accessibility. The objective of the study was to compare healthcare accessibility for older individuals since and during Covid-19 pandemic. The study was based on a sample of individuals from wave 8 Covid-19 add-on and wave 9 Covid-19-add-on of the Survey of Health, Ageing and Retirement in Europe (SHARE) in the period from 2020 till 2021. The sample size was 44043 respondents from 26 European countries. Descriptive statistics, Chi-square and McNemar test was performed. Overall, in 2021 there is a statistically significant decrease in proportion of respondents who forwent medical treatment (from 12% to 8%, in Latvia from 14,6% to 7%), postponed medical appointment due to Covid-19 (from 27% to 13%, in Latvia from 14,6% to 3,2%) and to whom appointment was denied by healthcare facility due to Covid-19 (from 5,3% to 4,5%, in Latvia from 6.9% to 3,3%) (p < 0.001). More often forwent medical appointment in 2021 was reported from females as well as respondents younger than 75 years, with good or very good self-rated health status and with less or no limitations due to health. Forwent medical treatment was reported more often from respondents with diabetes and hypertension. Results indicate that remote consultations are used more often than in 2020 (number of remote consultations varied between 1 and more than 100) and more often were reported by younger respondents (50-74 years). No significant association was found in self-rated health changes since and during Covid-19 pandemic for respondents who reported healthcare accessibility issues. As those issues were reported also by individuals with poor or fair self-rated health status and serious health conditions it can lead to a more rapid decline in health and functional abilities in the future.

**Key messages:**

- The situation with access to health care has improved during Covid-19, however further research is necessary to analyze how healthcare accessibility has affected health changes of older individuals.
- Telemedicine serves as a viable tool for maintaining patient care and lowering the risk of Covid-19 exposure to patients, however actions to improve digital skills for older individuals are needed.

